# Innovation technology opportunity identification of civil aircraft mechanical connections based on generative topographic mapping

**DOI:** 10.1371/journal.pone.0293309

**Published:** 2023-10-20

**Authors:** Lijie Feng, Huyi Zhang, Jinfeng Wang, Kuo-Yi Lin, Jinzhang Li

**Affiliations:** 1 Logistics Engineering College, Shanghai Maritime University, Shanghai, China; 2 Institute of Logistics Science and Engineering, Shanghai Maritime University, Shanghai, China; 3 China Institute of FTZ Supply Chain, Shanghai Maritime University, Shanghai, China; 4 School of Business, Guilin University of Electronic Technology, Guilin, China; 5 Aeronautical Manufacturing Technology Institute, Shanghai Aircraft Manufacturing Co., Ltd., Shanghai, China; China University of Mining and Technology, CHINA

## Abstract

In order to advance civil aircraft manufacturing to higher levels, there is an urgent need to identify technological innovation opportunities to help new technology development. This paper first analyses the current state of the research field and determines the topic. It preprocesses papers and patents within the research topic to obtain a base database. Then, the database is analyzed using the LDA (Latent Dirichlet Analysis) cluster analysis method. The TF-IDF (Term Frequency-Inverse Document Frequency) algorithm processes the data to obtain critical technical words. The abstracts of patents and papers are processed to construct a binary-based vector of technical keywords. The papers and patents are visualized in a two-dimensional space technology map by generative topographic mapping (GTM) to create a technology map to identify technology blank dots. The combination of technologies characterized by each technology blank dot is obtained by GTM inverse mapping. Finally, technology opportunities with a high probability of development are identified to achieve innovation opportunity identification. It also provides countermeasures for the research institution, enterprise, sector, and industry. After research and analysis, the future in the mechanical connection technology of civil aircraft is necessary to strengthen basic technology development and improve the study of intelligence, integration, and flexibility. Technology such as sensors and lasers can improve the precision and efficiency of mechanical connections.

## Introduction

Civil aircraft’s independent development is a concentrated expression of a country’s comprehensive strength. It is capable of driving steady growth in the national economy. It also generates significant industrial clusters and industrial radiation effects. As a result, many countries focus on supporting civil aircraft manufacturing as a strategic industry. As a complex system project, the civil aircraft project involves many technical areas in its manufacturing process, including cold processes, thermal processes, specialized processes, additive manufacturing, process equipment, and many other aspects. Among them, mechanical connection, the primary connection method for civil aircraft, accounts for more than 70% of civil aircraft structural connections. It is widely used in civil aircraft manufacturing and has an essential impact on quality, cycle time, and cost [1]. The continuous development of mechanical connection technology can effectively improve the efficiency and reliability of civil aircraft manufacturing.

Mechanical joining is the process and method of joining parts together using fasteners. It is characterized by reliable connections, simple processes, and repeated disassembly. In the study of mechanical connection technology, many scholars have researched the process, performance, and application of mechanical connections. The standard connection methods in the mechanical connection process are bolt connection [2], screw connection [3], rivet connection [4], etc. With the development of techniques, new types of connections, such as self-punching screw joints [5], electromagnetic riveting [6], automatic riveting [7], and interference fit connections [8], are widely used. Such development has dramatically improved the efficiency and quality of connections and extended the appropriate fasteners’ service life. Along with the promotion of new processes, new technologies, and the extensive use of new materials, much of the current research is focused on analyzing the performance of mechanical joining technology. For example, He et al. [9] investigated the mechanical properties of glued laminated timber beam-column connections with screws as fasteners. Lee et al. [10] investigated the mechanical properties of PFRP materials when used for single-bolt connections. Liu et al. [11] used composite fasteners as the research object and tested the pull-out performance of the fasteners. They optimized the relevant structural parameters based on the response surface method. In terms of specific applications, mechanical connections are widely used in the construction and manufacturing industries. Suzuki [12] carried out a parametric study of various stud and reinforcement sizes, configurations, and material properties to assess the mechanical properties of stud connections in reinforced concrete composite beams. Zhang [13] analyzed the joining process and methods for carbon fiber-reinforced polymers and metals in the aerospace, automotive, and marine manufacturing industries. Rakotondrainibe et al. [14] analyzed the coupling topology optimization of mechanical connections of automotive parts for mechanical system optimization for the weight reduction problem in automobiles.

Focusing on the civil aircraft manufacturing process, the continuous development, update, and iteration of mechanical connection technology can effectively improve the efficiency and reliability of civil aircraft manufacturing. In addition, with the increase in the proportion of composite materials used in civil aircraft and the increased requirements for quality and efficiency in civil aircraft manufacturing, mechanical connections are evolving towards high efficiency, high quality, long life, and high reliability. In this process, the technology, performance, and application of mechanical connection are the focus of current research and are of great interest. However, in the process of continuous updating and iteration of mechanical connection technology, predicting and grasping its technical development direction is a problem worth exploring. In this regard, it is necessary to identify innovation opportunities, grasp the development direction, and build an innovation path for mechanical connection technology to avoid the innovation risks caused by the blind and disorderly development of technology. On this basis, the efficiency of resource utilization is improved. In turn, we will achieve breakthroughs in mechanical connection technology, enhance civil aircraft manufacturing-related capabilities, and promote civil aircraft manufacturing to the high end.

This paper is structured as follows: Section 2 reviews the theoretical background of the main methods and techniques, including the current state of development of mechanical connectivity techniques, technological innovation opportunity identification, and generative topographic mapping. Section 3 explains the research thinking and the process of identifying technological innovation opportunities. Section 4 describes the application process using the example of mechanical connection technology for civil aircraft. Section 5 discusses the research institution, enterprise, sector, and industry levels. Finally, Section 6 concludes the paper, reviewing its limitations and recommending subsequent research.

## Literature review

### LDA-based text information mining

LDA (Latent Dirichlet Analysis) is a three-layer Bayesian topic model proposed by David Blei et al. [15] in 2003. It uncovers implicit thematic information in the text through unsupervised learning methods. LDA enables topic aggregation and extracts information from unstructured text. It is also a good representation of the semantic relationships between topic words. Therefore, the LDA topic model is becoming a powerful bibliometric method in text mining. With the development of machine learning, LDA is widely used. Basilio et al. [16] used LDA to analyze papers on domestic violence in the past fifty years, which greatly improved the efficiency of analysis; Gottfried et al. [17] used LDA to extract emerging topics in the research field and verified the availability of information; Joung et al. [18] used LDA for online review keyword recognition and achieved good results; Zhang et al. [19] used LDA to conduct technical evaluation and roadmap analysis in the blockchain field based on patent data, and predicted future development trends; Basilio et al. [20] used LDA in the field of public security to identify the characteristics of crime in specific areas and help law enforcement agencies develop strategies to combat crime. TF-IDF (Term Frequency-Inverse Document Frequency) is also a very important method in text information mining. Wang et al. [21] used TF-IDF for keyword extraction in scientific research. Alammary [22] improved TF-IDF to extract features from Arabic questions. The results show that the improved TF-IDF has higher accuracy.

Relevant scholars use LDA to mine text information to identify the main topic information in massive text. However, the accuracy of subject word extraction cannot be guaranteed. Based on this, we propose to use LDA to mine papers and patent data, obtain the main topic information, and improve the accuracy and efficiency of topic term extraction through TF-IDF.

### Identification of technological innovation opportunities

The traditional approach is based on qualitative analysis in the study of technology innovation opportunity identification. It relies mainly on the experience of domain experts. For example, Rahim et al. [23] argue that technology opportunity identification through experts can make the results of scientific research more creative in their transformation into marketable products. Moon et al. [24] conducted a technical opportunity identification by developing a new method called MORF-Vision (Morphological Vision of the Future). The advantage of the above approach is that it is based on the creative foresight of the expert in the area of expertise, which makes it possible to obtain insightful visions. The disadvantage is that the technological innovation opportunities identified are susceptible to the constraints of the domain expert’s knowledge and experience [25].

The identification of technological innovation opportunities is mainly based on quantitative methods. It first originated from the technology opportunity analysis of Porter et al. [26]. Later, Yoon et al. [27] used a patent-based semantic analysis method. Concrete technology opportunities were mined through text mining methods such as patent keyword analysis and SAO analysis. Song et al. [28] proposed a new method based on the F-term. It enables the classification of patent literature according to the technical attributes of inventions and, thus, the mining of new technological opportunities. Park and Yoon [29] identified technological innovation paths for target firms through patent classification and collaborative filtering methods. Wang et al. [30] proposed a morphological analysis method based on subject-action-object (SAO) to detect priority combinations in morphological matrices. Furthermore, technological innovation opportunities for dye-sensitized solar cells (DSSCs) were identified. Hyejin et al. [31] proposed a calculation method of patent analysis combined with user emotional value. It explores technical opportunities in heterogeneous fields based on user needs. Choi [32] proposed a patent-based technology innovation analysis method to identify potential technology opportunities. Yoon et al. [33] proposed a technology opportunity mining method combining SAO and anomaly detection. It is used to screen "outlier patents" for technological innovation opportunities. Jia et al. [34] constructed a multidimensional technology innovation map based on patent and literature information mining. It analyses the technological opportunities for CBM extraction in terms of technological development trends and potential innovation paths. Wang et al. [35] proposed a visualization method to support semantics by constructing a knowledge graph of similar patents. It provides a reference for companies to research technology opportunities early in business. Yoon [36] constructed a technology opportunity recognition dictionary by using a patent text mining method based on the development of the computer-aided system Tech Pioneer for technology opportunity discovery. Jeong et al. [37] proposed an opportunity mining approach based on topic modeling and sentiment analysis of social media data, which is used to identify opportunities for product innovation. Geum et al. [38] proposed a method based on crucial graph and index verification. They studied how to effectively interpret key graph results to identify new technological opportunities.

The above shows that scholars have conducted in-depth research on identifying technological innovation opportunities. However, most of the research data comes from patents, leading to some technological opportunities being forgotten. With the rapid development of deep learning in natural language processing, innovative technology identification can be used to identify technological blank dots in research areas using technology maps to analyze multidimensional data such as patents and papers. Identifying technological innovation opportunities can be more effectively achieved through blank dot analysis.

### Generative topographic mapping

Access to technology blank dots is a very effective means of identifying innovation opportunities. There are currently three main approaches regarding the mining of technical blank dots: principal component analysis (PCA), self-organizing mapping network (SOM), and generative topographic mapping (GTM). PCA and SOM are less explanatory of technology blank dots and rely on researchers to interpret them subjectively [39]. GTM is a probabilistic method proposed by Bishop to map multidimensional data Spaces to low-dimensional latent Spaces using Bayesian theory [40]. It can form objective network nodes and realize reverse interpretation. It also overcomes the shortcomings of previous methods in terms of poor objectivity and inadequate interpretation [41, 42]. Son et al. [43] proposed applying the GTM algorithm to technology blank dot identification and achieved good results. GTM has a significant advantage in technology for blank dot mining. The GTM algorithm allows visualization of data and technology blank dots to be discovered visually. Therefore, GTM can overcome the shortcomings of subjective recognition and interpretation techniques and solve problems in classification, clustering, visualization, etc. [44].

It facilitates a further and more objective interpretation of the meaning of the blank dots. Jun et al. [45] used GTM to identify the technology areas’ blank dots and conduct an in-depth analysis. Wu et al. [46] took the field of universal terrestrial radio access (UTRA) technology as the research object. They used standard and patent data to draw a patent map through the GTM algorithm to identify technical blank dots. Identifying potential standard-essential patents (SEPs) is achieved by mapping back to the original space to obtain the meaning of the blank dots. The results show that the GTM algorithm can achieve good results in technology innovation opportunity recognition. Feng Jian et al. [47] identified innovation opportunities through patent information. They use the GTM algorithm to draw technology maps and reverse map blank emerging technology points for effective identification. Teng Fei [48] visualized patent information through GTM and reverse mapped it to obtain technology opportunities. Experts also review the meaning of the blank dots to improve the objectivity and reliability of identifying innovation opportunities. Once GTM has identified a blank technology point, its development opportunity forecast is also significant. Yoon et al. [49] identified technology blank dots by mapping out patents in 3D printing through GTM. They use various link prediction methods to predict and compare the identified technical blank dots and compare them. Then, an accurate prediction method is used to obtain technical opportunities with a high probability of development. Liu et al. [50] migrate corresponding ideas via cosine similarity and link prediction for the target technology opportunities discovered based on GTM.

The identification of blank dots by the relevant scholars is limited to the interpretation of keywords. There is little on how keywords can be turned into practical innovation opportunities and how relevant industries can exploit them. Based on this, we propose a technological opportunity through GTM reverse mapping.

## Data and methods

### Analysis framework

**Fig 1:**
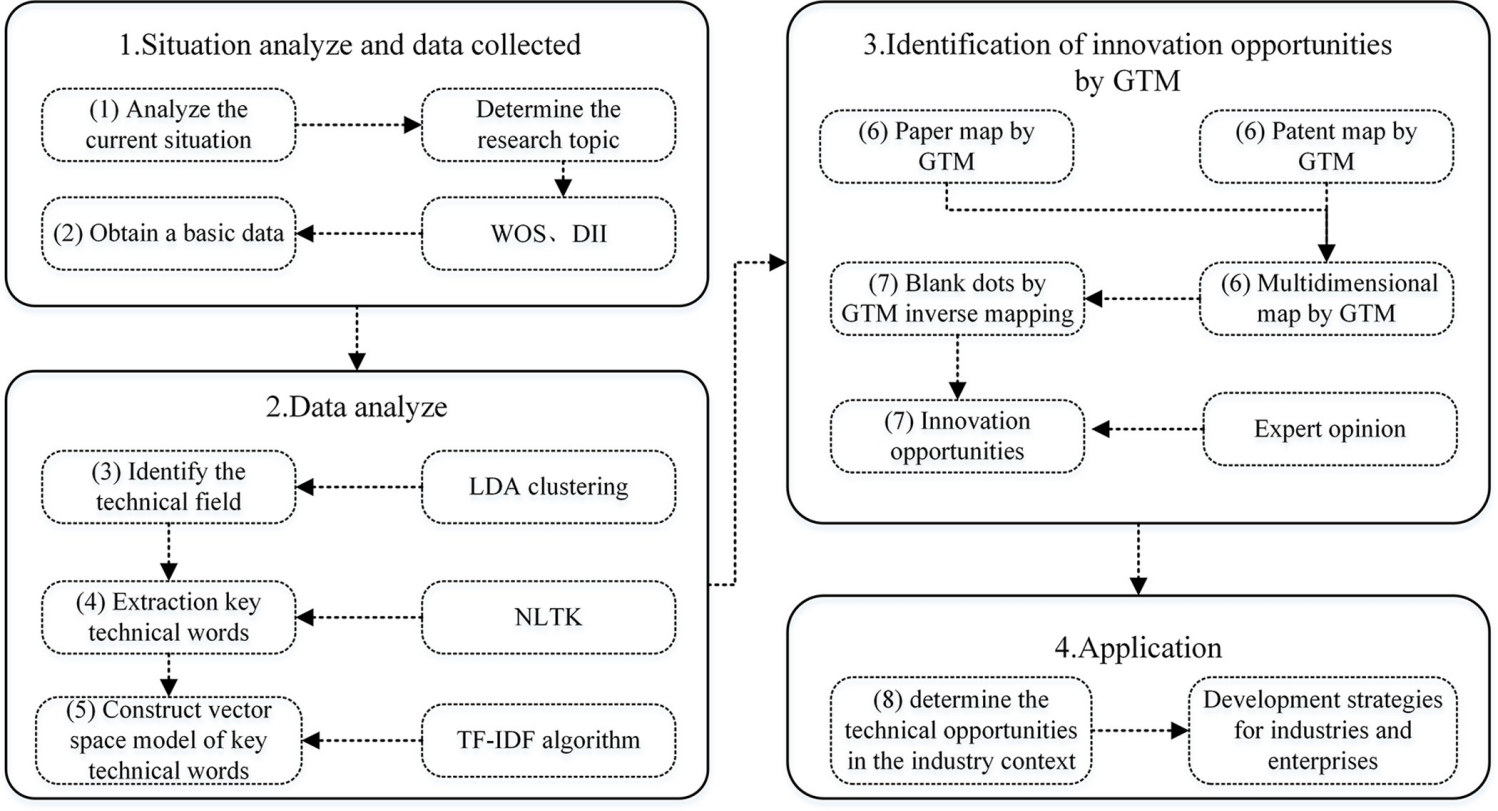
GTM-based analysis framework for identifying technological innovation opportunities.

The GTM-based analysis framework for identifying technological innovation opportunities consists of eight main steps, as shown in Fig 1. The first step is to analyze the current status of the research field and determine the research topic. The second step is to collect and preprocess the papers and patents on the research topic to obtain the paper and patent databases, respectively. The third step uses the LDA clustering topic model to analyze the database and identify the technical field. The fourth step extracts important keywords that represent the content of the entire document. The NLTK algorithm extracts the key technical words. The fifth step uses the TF-IDF algorithm to process the key technical words. It obtains the key technical word vector matrix of the paper and the key technical word vector matrix of the patent, respectively. The sixth step uses GTM to draw paper technology maps and patent technology maps. Then, a technical map based on patent and paper data is drawn to obtain technical blank dots. The seventh step is to obtain the combination of technologies characterized by the technical blank dots by GTM inverse mapping. The eighth step is to determine the technical opportunities with a high probability of development in the industry context. It provides development strategies for industries and enterprises and ultimately identifies innovation opportunities.

### Technology domain identification based on LDA topic models

LDA topic models are an important tool for text mining. The core of the LDA topic model is to view documents as probability distributions of implied topics. The topic is seen as a probability distribution of words. The document-to-topic ratio obeys a polynomial distribution. Topic-to-word follows a polynomial distribution. The parameters of that polynomial distribution obey a Dirichlet distribution. However, the LDA model may contain unimportant or irrelevant subject headings. It needs to be improved through domain expert screening or text preprocessing to enhance the accuracy of subject word extraction. Therefore, this paper applies the LDA topic model to identify research topics. It understands the distribution of technical topics as a whole. In turn, a more objective and accurate technical field is obtained.

### Key technical word extraction based on the TF-IDF algorithm

Technology domain distribution is determined through the LDA topic model. To further investigate technological innovation opportunities, this paper uses the TF-IDF (Term Frequency–Inverse Document Frequency) algorithm to analyze the technology domain and extract key technology words. The TF-IDF algorithm is a critical method in information retrieval [51, 52]. It is simple and effective. The term frequency (TF) indicates how often a keyword appears in a document. The inverse document frequency (IDF) reflects the prevalence of the keyword. When a word is more frequent in the document and has a lower prevalence, its TF-IDF value is higher. The TF-IDF balances term frequency and prevalence. It filters common words and retains essential words that provide more information. The author first processes the text for stemming extraction and lexical reduction before adopting the TF-IDF algorithm. The TF-IDF algorithm is then used to extract critical technical words. It can improve the accuracy and efficiency of subject lexical item extraction.

### Identification of technological innovation opportunities based on GTM

**Fig 2:**
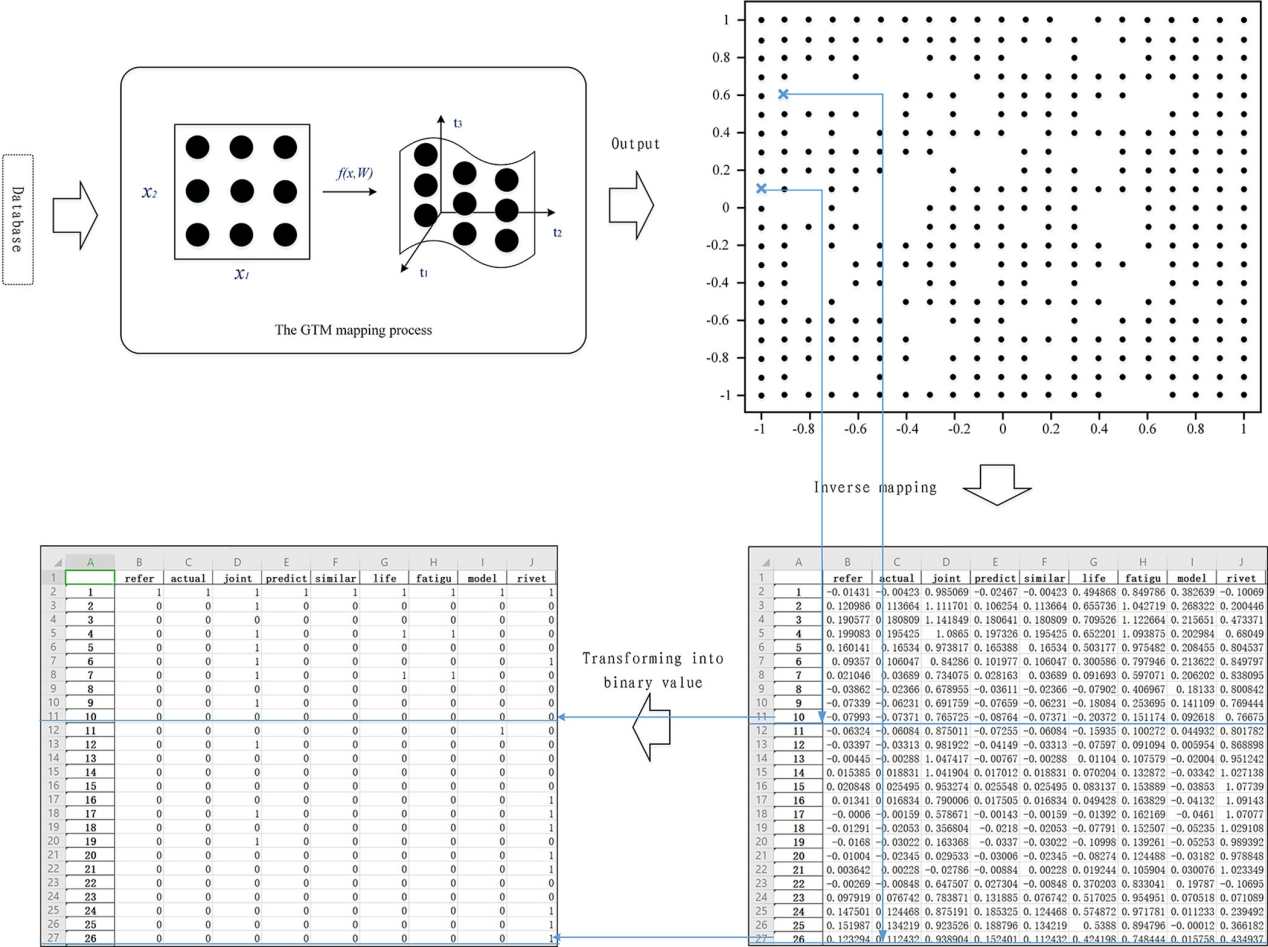
Example of GTM mapping process and inverse mapping.

Identifying technological innovation opportunities is increasingly becoming a hot research topic in academia. A technology map is an effective technical tool in technology innovation identification. A technical map maps raw high-dimensional data onto a low-dimensional grid through specific algorithms. Blank dots will appear if no corresponding technology combination is on the map. By analyzing and interpreting the points, technological innovation opportunities are identified. PCA, SOM, and GTM can provide all map technologies. PCA, SOM and GTM are used for blank technology mining, but PCA and SOM are more dependent on subjective interpretation by researchers. GTM is based on probability theory, can form objective network nodes, and is capable of reverse interpretation [40]. Therefore, this paper uses GTM to map the critical technology words extracted from the thesis and the patent. The above data are combined to draw a technology map under multidimensional data to obtain technology blank dots. It combines techniques characterized by inverse mapping to obtain blank dots. The GTM mapping process and reverse mapping are shown in Fig 2.

## Empirical study

### Analysis of current problems and data acquisition

In the aircraft assembly process, mechanical joining is an essential core technology. It is the key to improving aircraft assembly quality, efficiency, safety, and longevity. It plays an essential role in the production and development of aircraft. Therefore, identifying innovative technologies based on mechanical connections is significant for relevant industries to formulate R&D programs. The primary analysis data in this paper use SCI papers and patents, so the search expression is TS = (aircraft mechanical connection)) OR (aircraft fastener connections)) OR (aircraft riveting)) OR (aircraft bolt connection). We searched and downloaded the WoS (Web of Science) and Derwent data. WoS and Derwent are recognized as the most comprehensive databases of papers and patent data [53]. Among them, the time was set from 2000 to 2022, and the retrieval time was December 2022. A total of 355 papers and 3602 patents were obtained.

### Data preprocessing and technical topic identification

**Fig 3:**
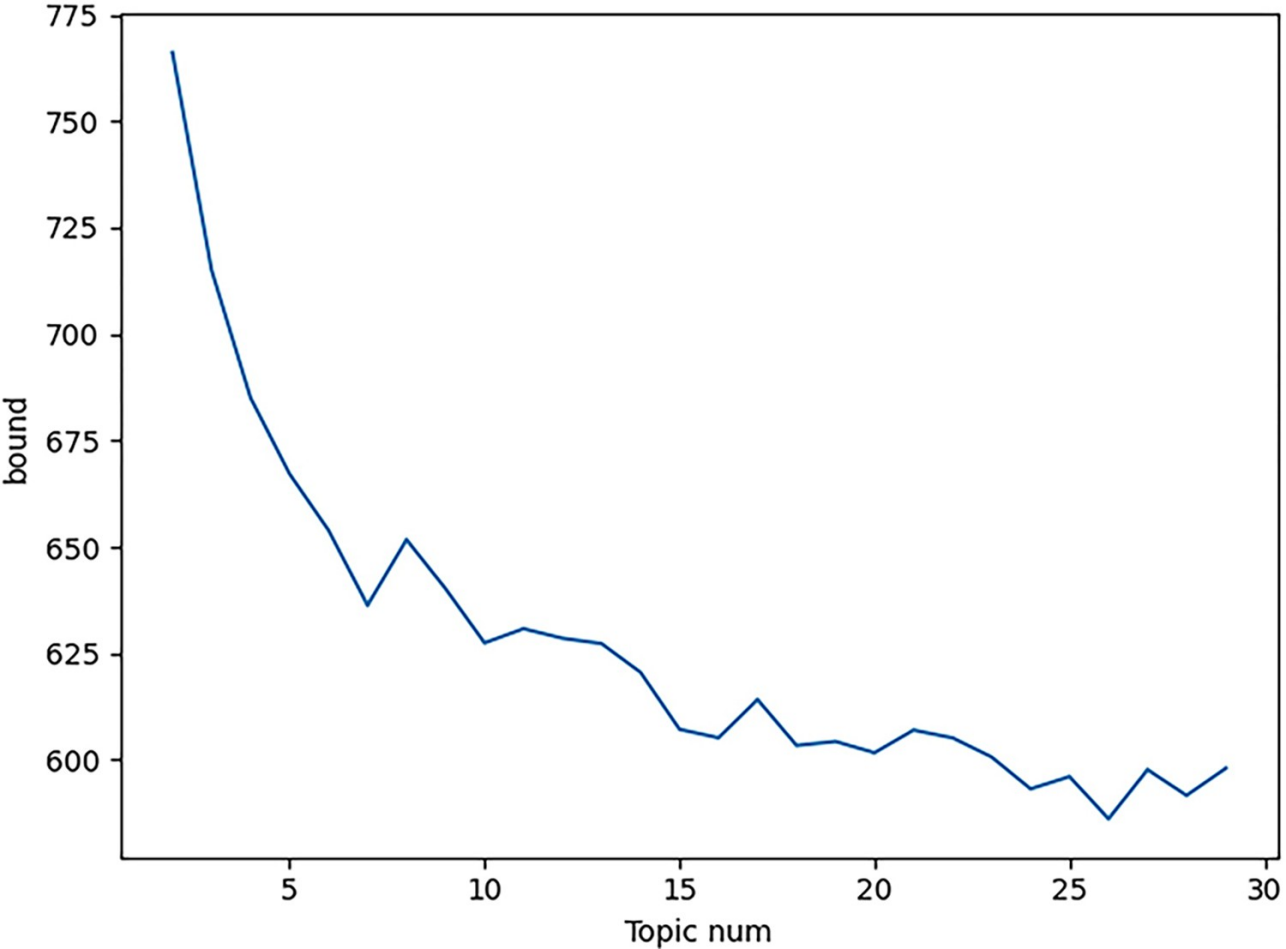
Patent clustering perplexity-topic number change graph.

The database based on the preprocessing, LDA was used to analyze the patents to bring up topics. The results of the analysis are shown in Fig 3.

Judging from the calculation results of the perplexity of the LDA model in Fig 3, when the number of topics is greater than 7, as the number of topics further increases, the rate of decline of perplexity slows down and gradually tends to be stable, and the marginal utility brought by further increasing the number of topics is not significant. Therefore, the number of patent clustering topics is determined to be 7. The results of these topics were named in conjunction with industry expert opinion, and the results are shown in Table 1.

**Table 1:** Results of patent analysis by LDA extraction of topics.

Number	Topic	Keywords
**1**	Device control	Device, sensor, control, air, fluid, signal, pressure, mechanical, chamber, cable
**2**	Electrical device	Unit, electrical, power, vehicle, electric, energy, circuit, element, mechanical, fuel, conductive, battery
**3**	Mechanical device	Device, arm, mechanism, machine, riveting, connected, shell, tool, wing, rod, cylinder, frame
**4**	Element assembly	Portion, structure, panel, support, unit, frame, assembly, fuselage, surface, device, wall, outer, seat, door, connecting
**5**	Bolt connecting	Plate, bolt, structure, hole, connecting, provided, connected, upper, model, utility, lower, body, fixing, groove, screw, rod
**6**	Rivet	Material, rivet, component, method, surface, layer, composite, fastener, structure, metal, fiber, portion, coating, hole
**7**	Connector	Module, connector, assembly, vehicle, housing, box, interface, board, base, mounting, light, tube, mechanical, electronic

**Fig 4:**
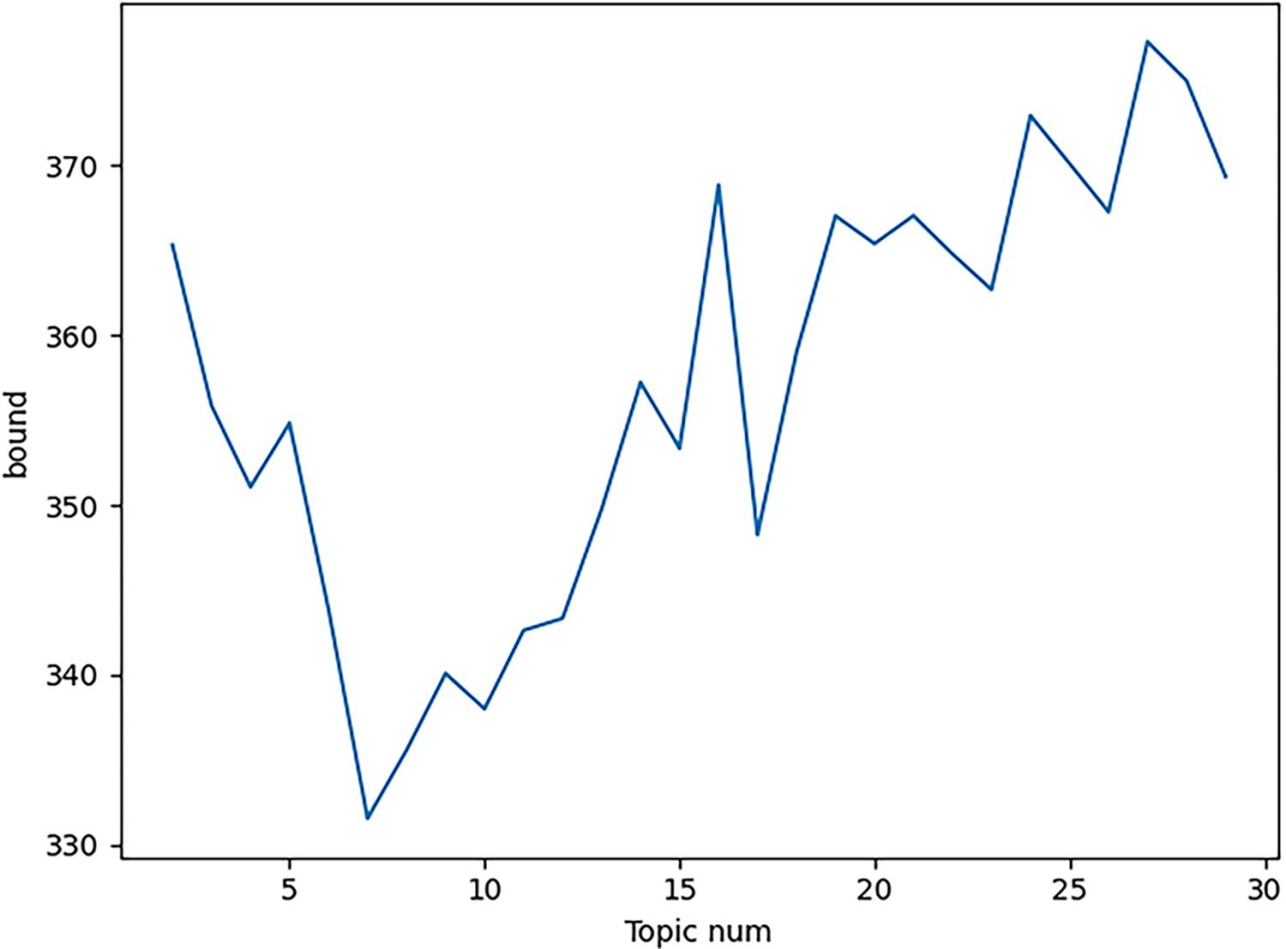
Paper clustering perplexity-topic number change graph.

Based on the preprocessing of data cleaning, LDA was used to analyze the papers to bring up topics. The results of the analysis are shown in Fig 4.

Judging from the calculation results of the perplexity of the LDA model in Fig 4, when the number of topics is equal to 7, the perplexity of the model is the lowest, and the perplexity-number of topics change graph of the clustering results turns a turning point here. Therefore, the number of paper clustering topics is determined to be 7. The results of these topics were named in conjunction with expert opinion, and the results are shown in Table 2.

**Table 2:** Results of papers analysis by LDA extraction of topics.

Number	Topic	Keywords
**1**	Mechanical property	Fatigue, stress, crack, hole, life, joint, growth, residual, specimen, model, riveted, fuselage, sheet, element
**2**	Material property	Composite, joint, material, adhesive, strength, bonded, failure, damage, mechanical, property, repair, laminate, fiber, carbon, patch, cfrp, bonding
**3**	Welding process	Laser, manufacturing, friction, joining, alloy, material, mechanical, cost, design, riveting, weight, stir
**4**	Connection property	Joint, load, riveted, lap, panel, stringer, crack, analysis, failure skin, structural, displacement, adhesive, tensile, stress, tolerance, strain, distribution, fatigue, factor, damage, influence
**5**	Drilling process	Hole, assembly, test, surface, force, vibration, quality, robot, material, panel, automatic, corrosion
**6**	Element analysis	Model, element, finite, plate, impact, connection, load failure, dynamic, contact, fuselage, experimental, joint, design, simulation, nonlinear, component, panel, fe(finite element), condition
**7**	Riveting process	Deformation, proposed, accuracy, assembly, model, process, quality, parameter, head, panel, force, compensation sheet, countersunk

As shown in Tables 1 and 2, the current research focuses on the connection process and mechanical properties. In this paper, based on identifying technical topics, the NLTK (Natural Language Toolkit) model was used to preprocess, split, stem extract, and lexically reduce abstracts on the Python (3.11.2) platform. It was supplemented by defining several stop words. NLTK [54] is a widely used Python natural language processing tool library, mainly used to complete word frequency statistics, word segmentation, part of speech tagging and other common tasks. The critical technical words with the highest TF-IDF values were extracted for each abstract using SciKit-Learn’s TF-IDF tool. Based on the extracted critical technical words, the abstract texts of patents and papers are processed to construct a binary-based vector of technical keywords. When the text contains the critical technical word, the corresponding element value is 1 and not included as 0. The matrix of patented key technical words (1433*3390) and paper key technical words (1185*343) were obtained, respectively.

### Identification of technological innovation opportunities

The technology development directions in mechanical connections were obtained through LDA cluster analysis. We used GTM to draw maps for technology blank dot analysis to analyze the development direction profoundly and to obtain technology opportunities. The input data are a technology word matrix of patent and paper data.

In this paper, based on the number of nodes to be overwritten [49], a 21*21-dimensional Gaussian function is chosen as the basis function, and the center of each function in the matrix is located in a 21*21 grid. The width of the function is set to 2, and the regularization parameter is set to 0.001. The technology word matrix is input into MATLAB’s GTM algorithm to build technology maps [55].

**Fig 5:**
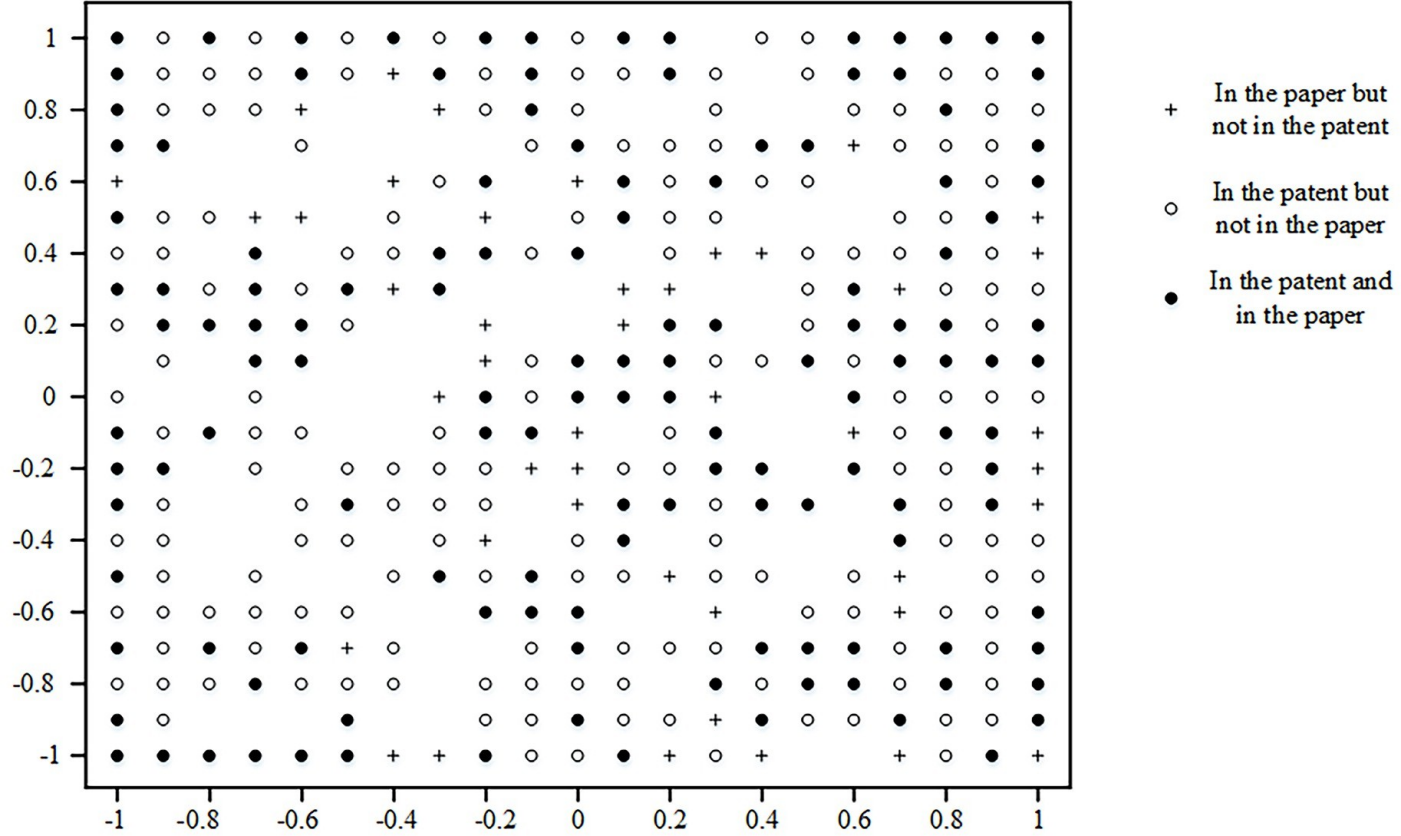
Aircraft mechanical connection technology map based on patent and paper data.

This paper draws a technical map based on the patent technology map combined with the paper data, as shown in Fig 5. Therefore, the technical map formed by the GTM algorithm based on patent and paper data differs from the previous technical map formed only by patent data. There are four types of technical maps formed by patent and paper data. In the diagram, a "+" indicates a technical point covered in a paper but not a patent. A "○" means a technical point covered in a patent but not a paper. "●" indicates a technology point covered in both a patent and a paper. The rest are blank dots.

GTM inverse mapping can be carried out to distribute each point in the raw data to obtain a combination of technologies characterized by the technical points. The probabilities corresponding to the identified technical points after reverse mapping are analyzed. A threshold is set to compare the possibilities; points more significant than this threshold are considered identified technical points. In this paper, the threshold is set to 0.2 by referring to the research, and the technical combination represented by each technical point is obtained. Enterprises focus on the technology process from theoretical research to production application and can explore the technical direction from papers without patents ("+").

The study provides research directions for enterprises to carry out technological innovation. Table 3 shows the technological innovation opportunities covered in the papers but not the patents. Each innovation opportunity involves several key technology words, which may be the future direction. For enterprises, they want to find the direction of technology development and pay more attention to the application of technology. The patent-free area can help enterprises explore new technology applications, promote better development of enterprises and formulate strategic goals.

**Table 3:** Opportunities for technological innovation are covered by papers but not by patents (part).

Number	Keywords
**1**	Joint, life, fatigue, model, rivet, stress, fe(finite element), lap, distribute, compose, layer
**2**	Life, fatigue, adhere, force, squeeze, load, bond, strength, structure, specimen, analysis, panel, control, metal, aluminum, alloy
**3**	Model, rivet, type, hybrid, force, fe(finite element), plate, elastic, join, crashworthiness, unit, turbine, engine, gas
**4**	Mechanical, device, element, second, unit, assemble, rivet, point, robot, dual
**5**	Rivet, point, cloud, robot, plan, mechanical, device, element, unit, control, assemble
**6**	Stress, sheet, strain, weld, fsw(friction stir welding), tensile, lap, residue, structure, panel, skin
**7**	Hybrid, force, failure, vibrate, element, finite, blade, rotor, turbine, case, engine, gas
**8**	Stress, hole, strain, residue, cold, repair, patch, intense, structure, panel, frame
**9**	Model, process, mater, manufacture, rotate, assemble, shaft, engine
**10**	Process, point, robot, plan, join, resource, optimal, locate, automatic, product, fixture, project, sand, polish, acquire, average, aeronaut, weight, effector, barrel, choice, maintain, device, second
**11**	Adhesive, test, composite, bond, structure, repair, patch, hole, body, shaft, main, frame
**12**	Model, point, robot, method, compensate, calibrate, posit, machine, optimal, dual, sample, accuracy, kinematic, base, coordinate, grid, device, second
**13**	Strain, load, composite, bond, hole, frame, assess, damage, repair, patch, dic(digital image correlation)
**14**	Test, eddy, structure, use, resist, corrosion, multi, hole, body, shaft, main
**15**	Vibrate, method, electric, inspect, connect, rotate, sleeve, shaft, rod
**16**	Hole, vibrate, method, inspect, connect, rotate, sleeve, shaft, rod
**17**	Model, assemble, method, propose, parameter, compensate, calibrate, position, align, machine, dual, pose, workspace, accuracy, kinematic, coordinate, tracker, orient, relate, mechanical, connect, element, second
**18**	Structure, ultrasonic, corrosion, magnet, detect, inspect, damage, monitor, wave, wing, manage, undamaged, connect, plate, mount, support, arm, rod, machine, unman
**19**	Sensor, detect, image, defect, probe, ec (eddy current), excite, plate, clamp, device, fix, upper
**20**	Process, material, hole, drill, assemble, composite, sensor, machine, tool, coat, fasten, connect, device, rotate, shaft, rod

**Fig 6:**
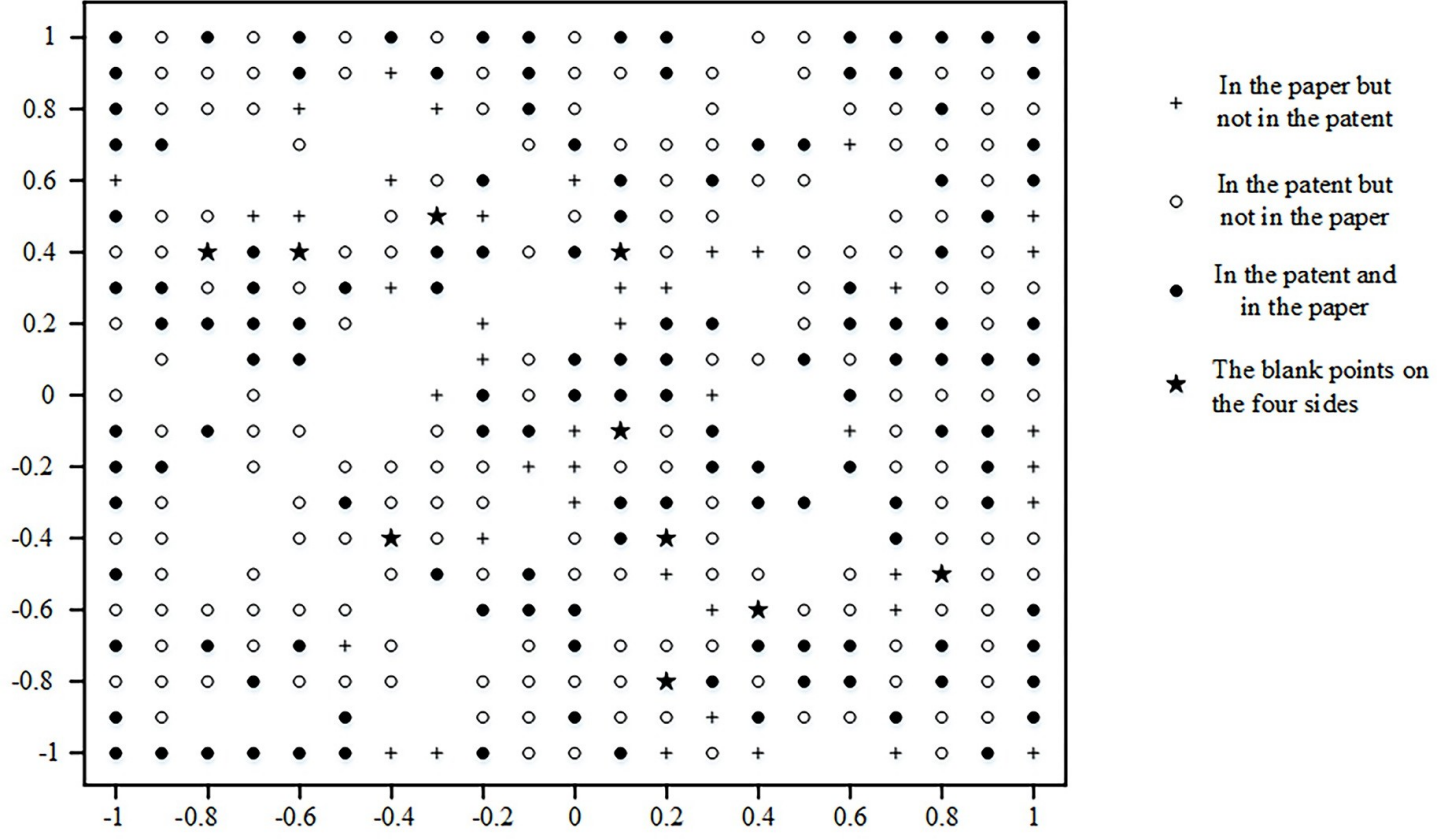
Map of emerging technologies for aircraft mechanical connections based on patent and paper.

Yoon et al. [49] found that in the technology map of GTM mapping, the blank spots adjacent to existing technologies have a greater probability of developing into the next phase of emerging technologies. At the same time, technology blank dots may be the key to future technology development and become the way forward. In the GTM mapping technology map, the blank dots on the four sides of the map are marked (indicated by "★"). They are used as future emerging technology points, as shown in Fig 6. This paper identifies ten technology blank dots geared toward aircraft mechanical connections. The critical technology terms they may contain are shown in Table 4.

**Table 4:** Opportunities for technological innovation in aircraft mechanical connections.

Number	Keywords
**1**	Joint, adhesive, hybrid, layer, bond, strength, lap, compose, fasten, aluminum, alloy
**2**	Joint, adhesive, hybrid, weld, load, tensile, bond, strength, fasten, panel, aluminum, alloy
**3**	Type, force, squeeze, failure, fe(finite element), composite, elastic, element, diameter, plastic, fracture, finite, brittle, shank, crashworthiness, blade, control, rotor, turbine, case, engine, gas
**4**	Stress, sheet, strain, weld, fsw(friction stir welding), load, tensile, lap, residue, structure, panel, skin
**5**	Stress, strain, residue, panel, cover, frame
**6**	Model, assemble
**7**	Model, process, material, assemble, shaft, engine
**8**	Process, dynamics, robot, join, panel, manufacture, rotate, shaft, engine
**9**	Model, process, material, weld, manufacture, rotate, assemble, shaft, rod
**10**	Hole, vibrate, method, inspect, connect, fix, sleeve, rod

This paper identifies ten technology blank dots geared toward aircraft mechanical connections. The critical technology terms they may contain are shown in Table 4. For example, the critical technical terms covered in Blank Technical Point 8 are process, dynamics, robot, join, panel, manufacture, rotate, shaft, and engine. It is possible to see significant innovation opportunities in the future for the use of robotics for mechanical connections in the manufacture of civil aircraft.

### Application analysis

A certain amount of technology has been developed in aircraft mechanical connections. However, the available technology still does not meet the actual development needs. Therefore, identifying technological innovation opportunities related to aircraft mechanical connections is urgently needed. Doing so will provide a clearer understanding of the direction of the development of mechanical connection technology in aircraft manufacturing. This will enable us to develop our next R&D plan and conduct technical research with our foundation. Combined with the technology blank dots identified in the previous section, innovative technologies can be provided for the future development of the companies involved.

In applied research, the first step is strengthening the overall basic technology and process mechanism research and development. As shown in Table 4, the key to future technological development is still the study of basic technology and fundamental processes. In particular, the influence of the relevant connection technology on stress-strain, structural strength, and fatigue life is a hot spot and focus of ongoing research in aircraft mechanical connections. Companies need to further strengthen the research on basic technologies and the development of technical processes if they are to have a prolonged effect.

Second, intelligent, integrated, and flexible research should be improved. In particular, regarding end-effector functionality, more attention should be given to applying technologies such as offline programming, motion simulation, and robot training. Combined with multi-sensor technology, these would give the end-effector the ability to perform state sensing, real-time analysis, autonomous decision-making, and precise execution. Technologies should be accumulated along with finding breakthroughs by focusing on nonproprietary literature.

Third, technologies such as sensors and lasers can improve the accuracy and efficiency of mechanical connections. In riveting, sensor technology can be used to improve riveting accuracy. For example, Boeing US9446444 uses multiple light sensors to detect the projectile’s position and control its speed. It provides precise control of interference during rivet installation. GEMCOR US6295710 and US9021677 use servo riveting to precisely control the applied riveting force using sensors on the presser foot.

Regarding development proposals, it is crucial to follow the patent trends of leading companies. Technical cooperation with research institutes and equipment suppliers should be strengthened. Collaborative R&D is a model that leverages the strengths of all parties involved to complete technological innovation jointly. It can avoid uncertainties in R&D, prevent risks and shorten the R&D cycle. Companies must strengthen cooperation and communication with suppliers, universities, and institutes. Through project cooperation and exchange, we can acquire the critical technologies needed and understand the progress of technological development. At the same time, with the rapid development of aircraft mechanical connection technology worldwide, relevant enterprises should strengthen their defensive patent layout strategy and find a new differentiated development direction. After achieving technology accumulation, technology with independent intellectual property rights is gradually formed.

## Discussion

Based on papers and patents, this study uses natural language processing, machine learning, and other methods to study mechanical connection technology in civil aircraft manufacturing. Based on the analysis above, the current research base and experimental conditions are considered, and the relationship between patents and papers is combined. The discussion is carried out at four levels: research institutions, enterprises, sectors, and industries, and relevant recommendations are made.

At a research institution level, investment in areas of technology where papers and patents are not available should be strengthened. The technology map’s blank dot represents when there is neither a patent nor a paper. This scenario indicates that this point is a technology blank dot, and neither patent placement nor paper research exists. For example, the keywords involved in Technology Point 243, which are not found in the paper or the patent, include “model” and “assemble.” The technology blank dot involves the study of theoretical models of the process. According to scholars [56, 57], research has shown that less research and practical exploration has been carried out on theoretical models of emerging technologies for mechanical connections. Examining this blank dot makes it possible to effectively identify technological innovation opportunities and predict the future direction of technology development. One is for technology blank dots where technology points already exist around them. It has a high probability of evolving into an emerging technology of the future and becoming the technology of the future. The technology point can be mapped backward to identify the keywords involved in the technology blank dot. These keywords are combined as an essential reference basis for future technology development. It determines the R&D value and development prospects of technology blank dots. The second is for blank technology dots where no surrounding technology point exists. This indicates that the point corresponds to a low level of maturity of the relevant technology, which is still in the primary research stage. It does not yet have the capacity for practical application and industrial diffusion. The development of the blank dot can be followed continuously.

From the enterprise level, planning papers without patents in the technical field exist. In the technical map, technical points exist in papers but not patents. This scenario indicates that this technology interests institutions and has produced results. However, patents do not yet exist for functional technologies, products, or services that meet specific needs. For example, keywords covered by Technology Point 69, for which some papers are not patented, include “robot,” “optimal,” “locate,” “automatic,” “fixture,” and “polish.” The technology blank dot involves research into the automation of mechanical connections. According to scholars [58–60], research on the mechanical connection of advanced processes exists in many theoretical studies. However, there are fewer real-life applications. The study of this technical point has a more significant reference value for developing enterprises. First, it helps companies plan the layout of new products and services. Product placement is achieved by analyzing the literature on the technology point to identify the potential for commercialization and to explore possible new products. Second, it helps companies to identify emerging areas. Based on the analysis of the commercialization potential of technology points, we forecast the direction of their commercialization, explore possible emerging areas and carry out relevant industrial layouts. Third, it helps develop a company’s R&D strategy by providing a reference for developing new technologies, processes, products, and services. It leads to targeted patent placement.

At a sector level, the focus is on areas of technology with patents but no papers. The technology map shows patents, but no papers exist for technology points. This scenario indicates that enterprises are concerned about this technology point and have already made patent arrangements, but there is no paper published yet. For example, the keywords covered by Technology Point 393, which has a patent without a paper, include “vibrate,” “method,” “defect,” “connect,” “fix,” “sleeve,” and “rod.” Technology blank dots relate to connecting assembly structures and equipment. According to scholars [61–63], research on mechanical connection equipment institutional innovation has many applications, but there is less theoretical research. Studying this technology point has a substantial leading role in the sector’s development. For enterprises in this sector, one can identify competitors’ technological strengths and weaknesses by analyzing existing patents. Based on this, they can select suitable areas for R&D and make their patent layout more strategic and competitive. They can also explore innovation and growth points by analyzing the limitations of existing patents. In addition, it promotes the generation of new patents to improve the added value of products and lead the direction of industry development.

At the industry level, there is strong support for technology areas where both papers and patents are available. In the technology map, technology points exist in both patents and papers. This scenario suggests that this technology point interests both companies and research institutions. They have conducted the layout of patents and published relevant papers, which may be a hot spot for current research. Examples of keywords covered by Technology Point 70, available in both papers and patents, include “joint,” “adhere,” “hybrid,” “load,” “tensile,” “strength,” “panel,” “aluminum,” and “alloy.” The technical points relate to studying mechanical properties and materials in fundamental theory. According to scholars [64–66], future developments in mechanical connections have been found to focus on the mechanical behavior and modeling of connection interfaces, special connection constructions, and applications. This technology point study is valuable to industries, enterprises, and research institutions. First, for the industry, investment can be increased around hot research areas. It also guides the direction of investment and the community’s layout to enhance the funding’s effectiveness and relevance. Then, the innovation capability of crucial technologies is effectively enhanced. Second, enterprises should focus on research hotspots to analyze competitors’ current research status and trends. At the same time, it should consider the direction of its patent layout to improve its core competitiveness. Third, the intrinsic links between current research hotspots can be analyzed for research institutions. The goal is to grasp the evolution of hotspots and predict future research frontiers.

## Conclusion

This paper focuses on the complex system engineering of civil aircraft manufacturing and is based on the field of civil aircraft mechanical connections. We extract technical blank dots based on papers and patents using machine learning methods such as natural language processing and GTM. It also identifies technological innovation opportunities in the industry and the company. It provides direction for technological development and helps companies gain an advantageous position in future international competition.

Regarding methodology, the data from papers and patents within the research topic are first collected and preprocessed. The database is analyzed using the LDA cluster analysis method. The TF-IDF algorithm processes the data to obtain critical technical words. Abstracts of patents and papers are processed to construct a binary-based vector of technical keywords. Technical mapping of papers and patents is conducted using GTM. Then, the two are compared, and a technology map based on patent and paper is created to identify technology blank dots. The combination of technologies characterized by each technology blank dot is also obtained by GTM inverse mapping. Finally, innovation opportunities are identified by identifying technology opportunities with a high probability of development in the industry context. The paper also provides recommendations for the research institution, enterprise, sector and industry.

In the application research, the analysis shows the following. First, we should strengthen basic technology research and development as a whole. Second, it is necessary to improve intelligent, integrated, and flexible research. Particularly concerning end-effector functionality, more attention is paid to applying technologies such as offline programming, motion simulation, and robot training. Third, technologies such as sensors and lasers can improve the accuracy and efficiency of mechanical connections. At the same time, enterprises should strengthen their defensive patent layout strategies and look for differentiated development directions. After achieving technology accumulation, we should gradually develop technology with independent intellectual property rights.

Regarding suggestions for responses, this paper is based on the relationship between thesis patents. The recommendations are relevant at four levels: the research institution, enterprise, sector, and industry. At the research institution level, there is a need to strengthen investment in areas of technology where neither papers nor patents are available. At the enterprise level, plans exist for technology areas with papers and no patents. We should focus on the technical fields with patents and no papers at the sector level. There should be strong support for technology areas where both papers and patents are available at the industry level.

Of course, some research blank dots in this paper will be improved upon in future research. First, this paper identifies only the areas of opportunity and directions for future innovation in mechanical connection technology for civil aircraft. No specific innovative technological solutions have been developed for application by enterprises. At the same time, the analysis of the current problems in the study is still based on experience and is not yet scientific. In the future, the current situation will need to be analyzed with the help of new tools to solve business problems more specifically and promote industry development.

## Supporting information

S1 Data (ZIP)
